# Arabidopsis ubiquitin ligase PUB41 positively regulates ABA-mediated seed dormancy and drought response

**DOI:** 10.1007/s12298-024-01526-6

**Published:** 2024-11-23

**Authors:** Avinash Sharma, Shalev Goldfarb, Dina Raveh, Dudy Bar-Zvi

**Affiliations:** 1 Department of Life Sciences, Ben-Gurion University of the Negev, Beer-Sheva, Israel; 2 Doris and Bertie Black Center for Bioenergetics in Life Sciences, Ben-Gurion University of the Negev, Beer-Sheva, Israel

**Keywords:** Arabidopsis, PUB E3 ubiquitin ligase, ABA signaling, Dormancy, Germination, Drought

## Abstract

Seed germination is a tightly regulated, non-reversible developmental process, and it is crucial to prevent premature germination under conditions that may not allow the plant’s life cycle to be completed. The plant hormone ABA is the key regulator of seed dormancy and inhibition of germination. ABA is also involved in the plant response to drought. Here we report on the involvement of *Arabidopsis thaliana PUB41*, encoding a U-BOX E3 ubiquitin ligase, in regulating ABA signaling, seed dormancy, germination, and drought resilience. *AtPUB41* is expressed in most vegetative and reproductive tissues. AtPUB41 protein is localized in the cytosol and nucleus. *pub41* T-DNA insertion mutants display reduced seed dormancy, and their germination is less inhibited by exogenous ABA than seeds of wild-type plants. *pub41* mutant plants are also hypersensitive to drought. ABA induces *AtPUB41* promoter activity and steady-state mRNA levels in the roots. Our data suggest that *AtPUB41* is a positive regulator of ABA signaling.

**Supplementary Information:**

The online version contains supplementary material available at 10.1007/s12298-024-01526-6.

## Introduction

As sessile organisms, plants are continuously exposed to fluctuating environments and have evolved mechanisms that modulate their growth and development in response to environmental cues. The plant hormone ABA is a key player in modulating these developmental processes, from the breaking of seed dormancy for germination (reviewed in Jones 2016), to the response to abiotic stresses, including drought (Ma et al. 2018).

ABA signaling pathways have been elucidated: ABA binds to the soluble receptors RCAR/PYR/PYL, which then bind and inhibit PP2C protein phosphatase. PP2C inactivation leads to the activation of SnRK protein kinase that phosphorylates and activates downstream transcription factors, leading to an alteration in gene expression conducive for coping with global environmental changes (Tang et al. 2015). Moreover, the ABA response mechanism involves a change in the cellular proteome, resulting in specific changes in protein translation and degradation. The Ubiquitin-Proteasome System (UPS) is a central mechanism for controlled protein degradation in all eukaryotes including plants (Vierstra 2009). A large part of the plant genome, about 5% in Arabidopsis, encodes UPS components, reflecting its pivotal role in maintaining proteome homeostasis. The majority of these genes (> 1400 in Arabidopsis) encodes E3s that specifically recognize and ubiquitinate target proteins (Vierstra 2009). The UPS has a major role in ABA signaling (Yu et al. 2016). For example, the protein levels of ABA receptors, PP2A proteases, and ABA-regulated transcription factors are controlled by ubiquitination-mediated degradation by the 26S proteasome (Yu et al. 2016).

Here, we present the role of the Arabidopsis U-box E3 ligase *PUB41.* AtPUB41 is a member of the Plant U-Box (PUB) family of E3s, possessing a U-box domain that binds the E2 ubiquitin-conjugating enzyme and several repeats of the Armadillo (ARM) motif that bind the proteins targeted for proteasomal degradation (Trujillo 2018). PUBs have a major role in diverse biological processes, including development, immunity, and response to abiotic stress (reviewed by Vierstra 2009; Trujillo 2018). Here we show that the stress hormone ABA enhances the promoter activity of *AtPUB41* and the steady-state levels of this gene transcript. Furthermore, we show that* AtPUB41* is important for seed dormancy, germination, and the drought response: seeds of *pub41* mutants show reduced ability to maintain dormancy, and their germination is less sensitive to exogenous ABA. In addition, *pub41* T-DNA mutant plants are less resilient to drought than wild-type plants.

## Materials and methods

### Plant material

Homozygous *Arabidopsis thaliana* ecotype Columbia was used in this study. T-DNA insertion lines *pub41-1* (SALK_099012) and *pub41-2* (SALK_142012) were obtained from the Arabidopsis Resource Center, Columbus, Ohio. The presence of T-DNA insertion was confirmed by Polymerase Chain Reaction (PCR) using T-DNA and gene-specific primers, further confirmed by Sanger sequencing of the PCR product. Primers are listed in Supplementary Table S1.

### Plant growth conditions

Plants were cultivated at 22 °C, with a relative humidity of 50%, following a circadian cycle of 12 h light and 12 h dark. Seeds were surface sterilized and stratified at 4 °C for at least 4 days before they were sown on solid 0.5 × Murashige and Skoog (MS) + 0.5% (w/v) sucrose or in pots as described previously (Adler et al. 2017). Post-germination, application of ABA was performed by transferring with forceps plate-grown 7-days-old seedlings to Petri dishes containing Whatman No. 1 paper soaked in 0.5 × MS and with the indicated concentration of ABA. Plates were then incubated for 8 h in the light at room temperature.

### Seed germination

Surface-sterilized cold-treated seeds were sown in Petri dishes containing 0.5 × MS and 0.7% agar, and the indicated ABA concentrations. Plates were incubated at 22 °C in a 12 h light/12 h dark regime. Radicle emergence was assessed under a dissecting microscope. Seeds that developed ≥ 1 mm long radicles were scored as germinated.

### Drought tolerance

Plants were grown for 3 weeks in pots containing equal amounts of potting mix under non-stress conditions. Water was then withheld, and plant wilting and drying were visually followed daily. Upon wilting of the plant of the sensitive genotypes (approximately 2 weeks), both wild-type and mutant plants were rewatered, and plant survival was visually scored 4 days later.

### Transcript levels

RNA isolation, cDNA synthesis, primer design, and RT-qPCR assays for determining relative steady-state transcript levels were performed as previously described (Maymon et al. 2022). Primers are listed in Supplementary Table S1.

### Plasmid construction, plant transformation, and transient expression

The 35S::PUB41-eGFP plasmid was constructed by amplifying the DNA sequence encoding full-length PUB41 with primers containing linkers with the *Nco*I and *Pst*I restriction sites. This sequence was subcloned into the respective restriction sites of the pGA-eGFP3 vector (Maymon et al. 2022) in frame with the sequence encoding eGFP.

To construct *AtPUB41::GUS* expressing lines, a 1440 bp DNA fragment upstream to the start codon of the PUB41 gene was amplified using the primers listed in Supplemental Table S1 and genomic DNA of wild-type Arabidopsis plants. The amplified *AtPUB41* promoter DNA fragment was sub-cloned into the *Pst*I and *Eco*RI restriction sites of pCAMBIA 1391Z upstream to the GUS encoding sequence.

Plasmids were verified by DNA sequencing and introduced into *Agrobacterium tumefaciens* GV3101, and wild-type Arabidopsis plants were transformed by the floral dip method (Clough and Bent 1998). Homozygous transformants were isolated by selection on plates containing growth medium supplemented with hygromycin. Primers are listed in Supplementary Table S1.

Transient expression in *Nicotiana benthamiana* by leaf infiltration of *Agrobacterium tumefaciens,* and detection of the expressed proteins by fluorescence microscopy were performed as described by Eisner et al. (2021).

### GUS staining

Plant tissues, after treatment, were fixed into acetone chilled at - 20 °C, and GUS staining was performed as described (Jefferson 1987). Images were taken using a dissecting microscope equipped with a Dino-Eye: AM7025X Edge Series 5MP Eyepiece Camera.

### Statistical analysis

All experiments were carried out in at least 3 biological repeats. Data are expressed as average ± SE. Statistical significance was determined using Tukey’s HSD post hoc test and the Student’s t-test.

## Results and discussion

### Domain structure and cellular localization of AtPUB41

**Fig. 1:**
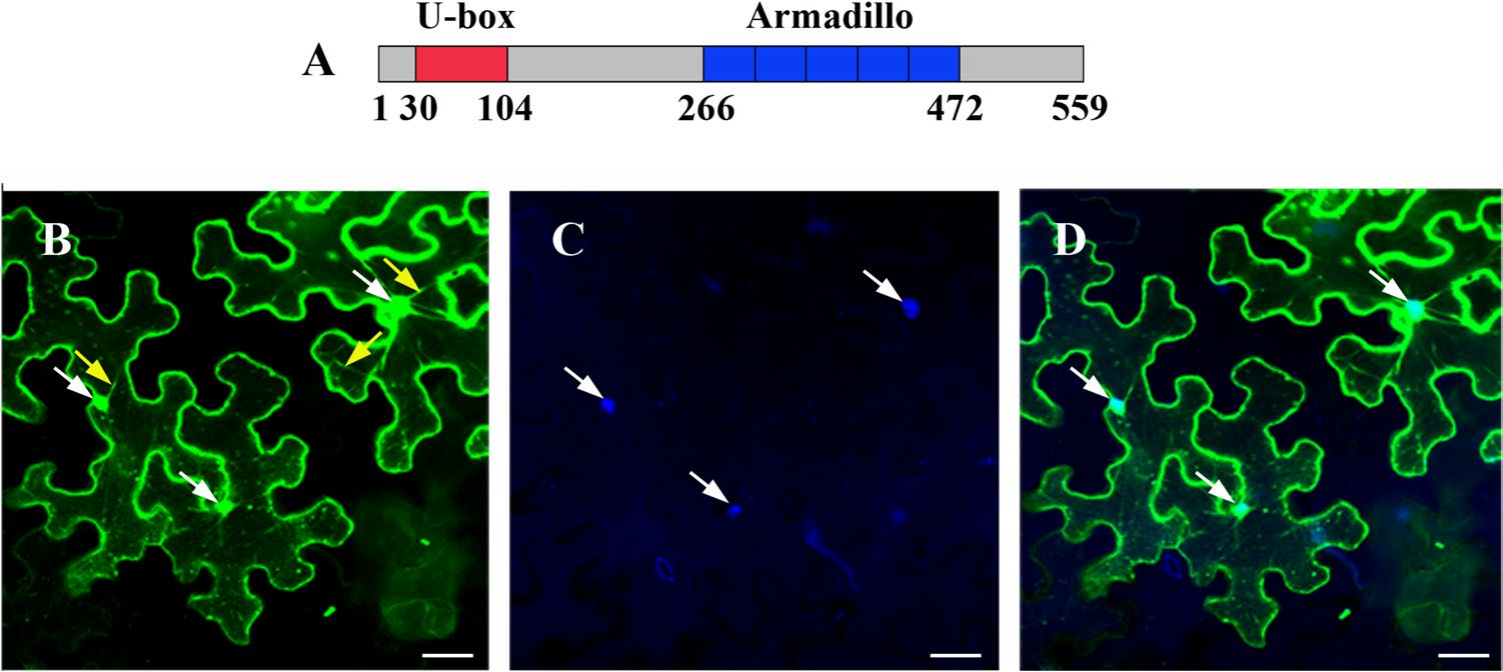
Domain organization and subcellular localization of AtPUB41. **A**. Diagram presenting the linear amino-acid sequence of AtPUB41 and functional motifs. Numbers represent the respective amino acids. **B**–**D**. Subcellular localization of AtPUB41. Leaves of *N. benthamiana* were infiltrated with Agrobacteria harboring *35S::AtPUB41-eGFP.* Lower epidermis peels were prepared and counterstained with the DNA marker DAPI, and GFP fluorescence (**B**) and DAPI (**C**) were analyzed by confocal microscopy. (**D**), merged image of (**B**) and (**C**). White and yellow arrows point at nuclei and transvacuolar cytoplasmic strands, respectively. Scale bar = 50 µm

The Arabidopsis *PUB41* gene (At5G62560) is an intronless gene that encodes a 559 amino-acid long E3 of the Plant-U-box (PUB) family. AtPUB41 has ubiquitin ligase activity (Wiborg et al. 2008). Motif analysis of the amino acid sequence of AtPUB41 indicates a U-box motif at residues 30–104, and a cluster of 5 ARM motifs located between residues 266–305, 307–346, 348–388, 390–427 and 428–472 (Fig. 1A).

To assess the subcellular localization of the AtPUB41 protein, we transiently expressed AtPUB41-eGFP in leaves of *Nicotiana benthamiana.* Lower epidermis peels were counterstained with the DNA binding stain DAPI (4′,6-diamidino-2-phenylindole), and cells were examined by confocal fluorescence microscopy. AtPUB41-eGFP was detected in the nucleus and the cytoplasm (Fig. 1). The cytoplasmic localization of AtPUB41 is supported by its presence in transvacuolar cytoplasmic strands (Fig. 1B), and the nuclear localization is demonstrated by colocalization of the AtPUB41-GFP signal with that of the DNA fluorescent stain (Fig. 1D). The NLS mapper (Kosugi et al. 2009) supports the nuclear import of AtPUB41, indicating three presumptive nuclear localization signals (NLSs) at residues 3–32, 165–194, and 256–287, with scores of 3.7, 3.5, and 3.2, respectively, supporting our experimental demonstration of its nuclear localization.

### Promoter activity of *AtPUB41*

**Fig. 2:**
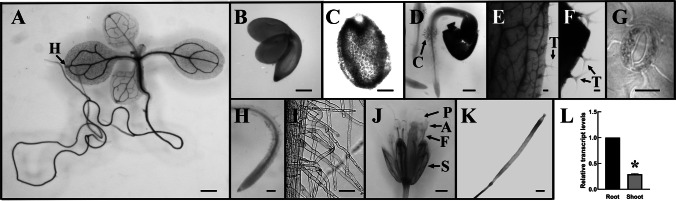
Tissue specificity of *AtPUB41* expression. (**A**–**K**) Arabidopsis plants expressing *GUS* reporter gene from the *AtPUB41* promoter stained for GUS activity. **A**. 8-days old seedling. The arrow marks the hydathode (H). **B**. Embryo rescued from imbibed seed. **C**. Endosperm cells in imbibed seed. **D**. 36-h-old Arabidopsis seedling. Arrow points to promoter activity in collet region root hairs (C). **E** & **F**. Sections of fully expanded leaves. Arrows point to trichomes (T). **G**. Stomata. **H**. Root tip and root meristem zone of the primary root. **I**. Mature root hairs of the maturation zone. **J**. Open flower: A, anther; F, anther-filament; P, petal; S, sepal; **K**. Silique. Bars: A, B, C, E, F, H, I, 0.1 mm; G, K, 0.05 mm; D, J, 0.25 mm. L. Relative *AtPUB41* transcript levels. The roots and shoots of 10-days-old wild-type seedlings grown on 0.5 × MS without supplements were harvested, RNA was isolated, and PUB41-transcript levels were determined, by RT-qPCR using primers #8, 9, 14 and 15 (Table S1). Data showing mean ± SE of three repeats. Asterisks denote statistical significance as determined by a two-tailed paired student’s t-test (*p* ≤ 0.002)

Only limited expression data for *AtPUB41* is available as the AT5G62560 locus was not included in the Affymetrix ATH1 microarrays. Therefore to examine the activity of the *AtPUB41* promoter, we transformed Arabidopsis plants with a plasmid encoding the β-glucuronidase (GUS) reporter gene driven by the *AtPUB41* promoter (*AtPUB41::GUS*), comprising 1440 bp upstream of the translation start codon. Plants of three independent homozygous lines were analyzed. Under controlled optimal growth conditions, the *AtPUB41* promoter was predominantly active in cotyledons, leaf vascular tissue, petioles, hydathodes, axillary buds, stems, and roots (Fig. 2A). High promoter activity was observed in embryos (Fig. 2B) and endosperm (Fig. 2C) of imbibed seeds. *AtPUB41* promoter activity was detected in fully expanded leaves (Fig. 2E–G), where, in addition to vascular tissue, notable expression was detected in trichomes and stomata guard cells (Fig. 2E–G). The *AtPUB41* promoter activity was also present in the root hairs of the collet (Fig. 2D) and root maturation zone (Fig. 2I). In mature plants, promoter activity was detected in sepals, pistils, anther filaments, and mature siliques (Fig. 2J–K). In contrast, promoter activity was not detected in the root elongation zone, mature anthers, or petals (Fig. 1D, H, J). Moreover, our RT-qPCR analysis of 10-days-old seedlings revealed a higher expression of *AtPUB41* in roots than in shoots, indicating a role for PUB41 in root tissue (Fig. 2L).

### *pub41* T-DNA insertion lines

**Fig. 3:**
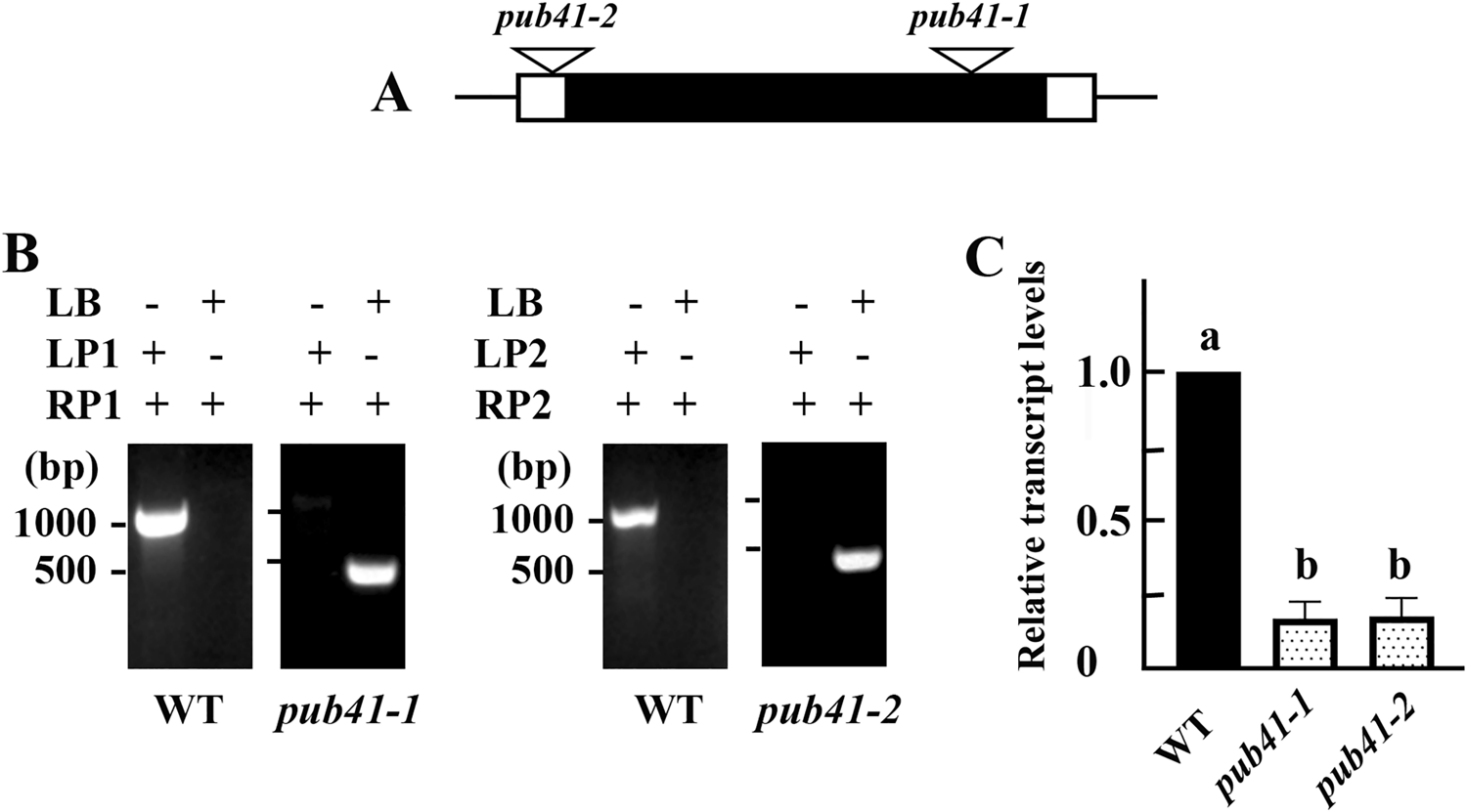
Molecular characterization of *AtPUB41* T-DNA insertion lines. **A** Diagram of the *AtPUB41* gene. Transcribed sequences are shown as boxes, where the black region marks the protein-encoding sequence, and the white regions mark 5′ and 3′ UTRs. T-DNA insertion sites of the respective mutants are marked by triangles. **B** Verification of the T-DNA insertion loci by PCR. Genomic DNA from the indicated genotypes was amplified using the T-DNA left border (LB) primer and site-specific left and right primers (LP1, LP2, and RP1, RP2) as indicated. **C** Steady-state levels of *AtPUB41* transcripts in 5-days-old seedlings of wild-type (WT) and *pub41* mutants. The data represent the mean ± SE of three independent experiments. Bars with different letters represent statistically different values by ordinary one-way ANOVA, Tukey’s multiple comparison test (*p* < 0.001)

Two *pub41* T-DNA insertion mutants were obtained from the Arabidopsis stock center, Columbus, Ohio. SALK_099012C and SALK_142330 are designated as *pub41-1* and *pub41-2*, respectively (Fig. 3A). The mutants were verified by PCR analysis using gene-specific and T-DNA border primers (Supplemental Table S1), as described earlier (O’Malley et al. 2015). Only the *pub41-1* and *pub41-2* lines yielded a product of ~ 500 bp with the LBb1.3 and RP primers (Fig. 3B). The amplified PCR product was subsequently used for Sanger sequencing to identify the precise location of the T-DNA insertion. The sequencing results revealed that the T-DNA inserted within the protein-encoding region (+ 1505 bp downstream from the start codon) in *pub41-1,* and within the 5′ untranslated region (UTR) (-69 bp upstream of the start codon in the *pub41-2* mutant (Fig. 3A). RT-qPCR analysis using primers #8, 9, 14 and 15 (Table S1), further confirmed that the transcript levels of *AtPUB41* in both mutants are less than 18% that of the wild-type plants (Fig. 3C).

### *pub41* mutants are hypersensitive to drought

**Fig. 4:**
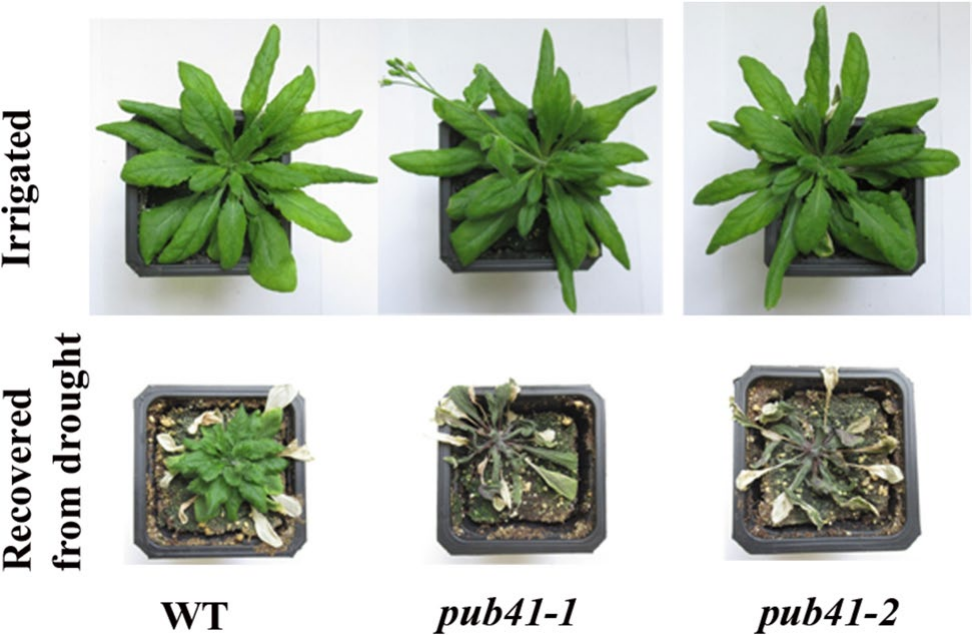
*pub41* mutant plants are more sensitive to drought than wild-type plants. Wild-type (WT) and *pub41* mutant plants were grown in pots for 6 weeks before water was withheld for 12 days. Water was supplied only after the plants were fully wilted and the plants were imaged 4 days later in the recovery period

We screened 25 homozygous T-DNA mutants from the Arabidopsis Stock Center, that have T-DNA inserted into genes encoding E3s, for changes in drought resilience (see Methods). Our original screen to identify UPS-associated genes that affect drought resilience led to the identification of 3 PUB genes*, AtPUB8, AtPUB46,* and *AtPUB41.* Mutants were verified as described (O’Malley et al. 2015) and Fig. 3. The involvement of each of these genes was confirmed using a single T-DNA insertion mutant (Adler 2011). To further confirm the drought-hypersensitive phenotype of the *pub41* mutant and to rule out the possibility that the drought hypersensitivity resulted from a positional effect, we repeated the water-withholding experiment using two independent* pub41* mutants. Figure 4 shows that both *pub41-1* and *pub41-2* mutants are hypersensitive to drought, and under the protocol applied, they failed to recover from water-withholding stress, in contrast to the wild-type plants. These results show that *AtPUB41* is essential for Arabidopsis drought stress resilience, and that *Atpub41* mutants are impaired in their ability to adapt to stress conditions.

Ubiquitin ligases belonging to the PUB family are recognized for their involvement in the plant response to abiotic and biotic stresses (Trujillo 2018, 2021; Vierstra 2009). Interestingly, *pub* mutants affected in drought resilience are both hyper- or hypo-sensitive: single gene mutants of the Arabidopsis paralogs *AtPUB46* and *AtPUP48* (Adler et al. 2017), and the *Atpub8* mutant (Adler 2011) are drought hypersensitive whereas Arabidopsis *Atpub18*, *Atpub19*, *Atpub23*, and *Atpub24* mutants exhibit increased drought tolerance compared with wild-type plants (Seo et al. 2012). Also, the rice *OsPUB7* knockout mutant and the *Ospub41* suppression mutant, and *Ubi:RNAi-OsPUB41* knock-down lines showed enhanced drought resilience (Kim et al. 2023; Seo et al. 2021). (Despite sharing a common gene name *OsPUB41* is not orthologous to *AtPUB41,* but shows the highest similarity to *AtPUB20* and *AtPUB21*).

### Seed germination of *pub41* mutants shows reduced ABA inhibitory effects

**Fig. 5:**
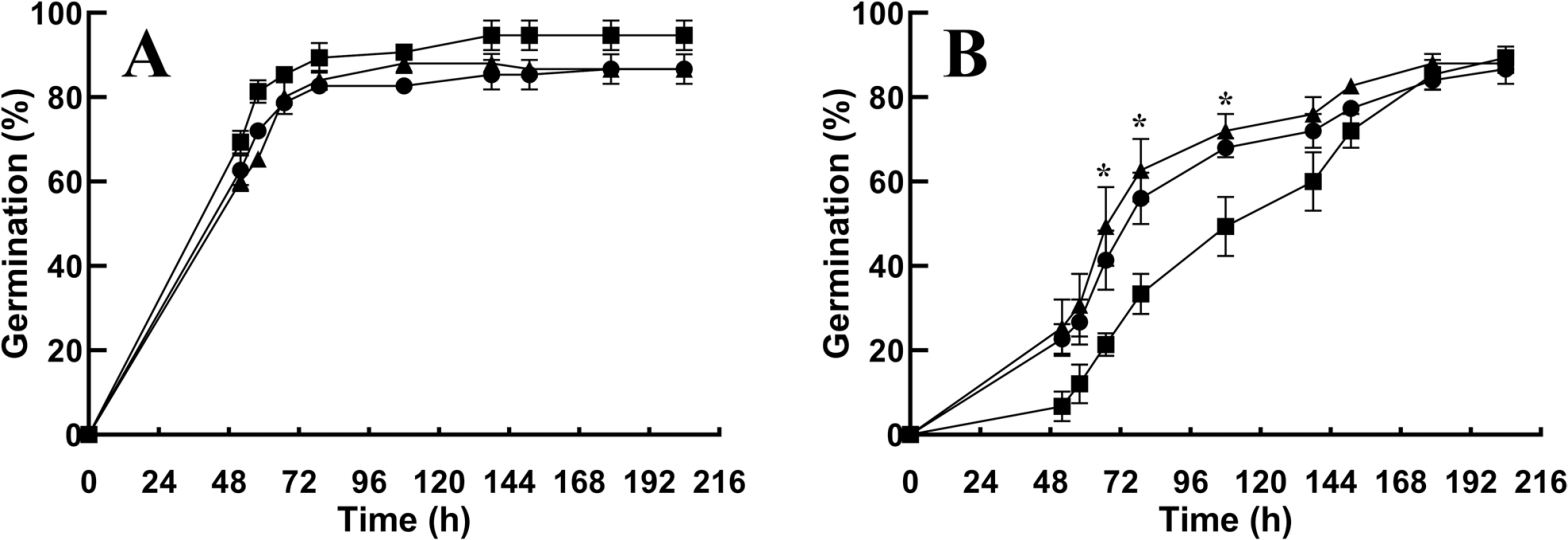
Germination of *pub41* mutants is less sensitive to ABA inhibition. Surface sterilized cold-treated seeds of the wild-type (WT) (■), *pub41-1* (▲), and *pub41-2* (●) genotypes were plated on agar-solidified 0.5 × MS 0.5% sucrose medium without (**A**) or with 2.5 µM ABA (**B**). Germination was scored at the indicated times. Data represent means ± SE of three independent biological repeats. Asterisks mark significant differences between the WT and mutant seeds according to Student’s unpaired t-test (*p* < 0.05)

In addition to drought, ABA is also the primary hormone that ensures seed dormancy and represses germination (Bewley et al. 2013; Ma et al. 2018). We therefore examined the impact of ABA on germination of seeds of the *pub41* mutants. Under normal growth conditions, the germination of *pub41* mutants was similar to that of wild-type plants (Fig. 5A). However, the germination of seeds of the *pub41-1* and *pub41-2* mutant lines displayed reduced sensitivity to ABA inhibition compared with wild-type plants (Fig. 5B). For example, 52 h following plating on growth medium containing 2.5 μM ABA, 25.3% and 22.7% of seeds of the *pub41-1* and *pub41-2* mutants germinated compared with only 6.7% of the wild-type seeds.

### Seeds of *pub41* mutants show reduced dormancy

**Fig. 6:**
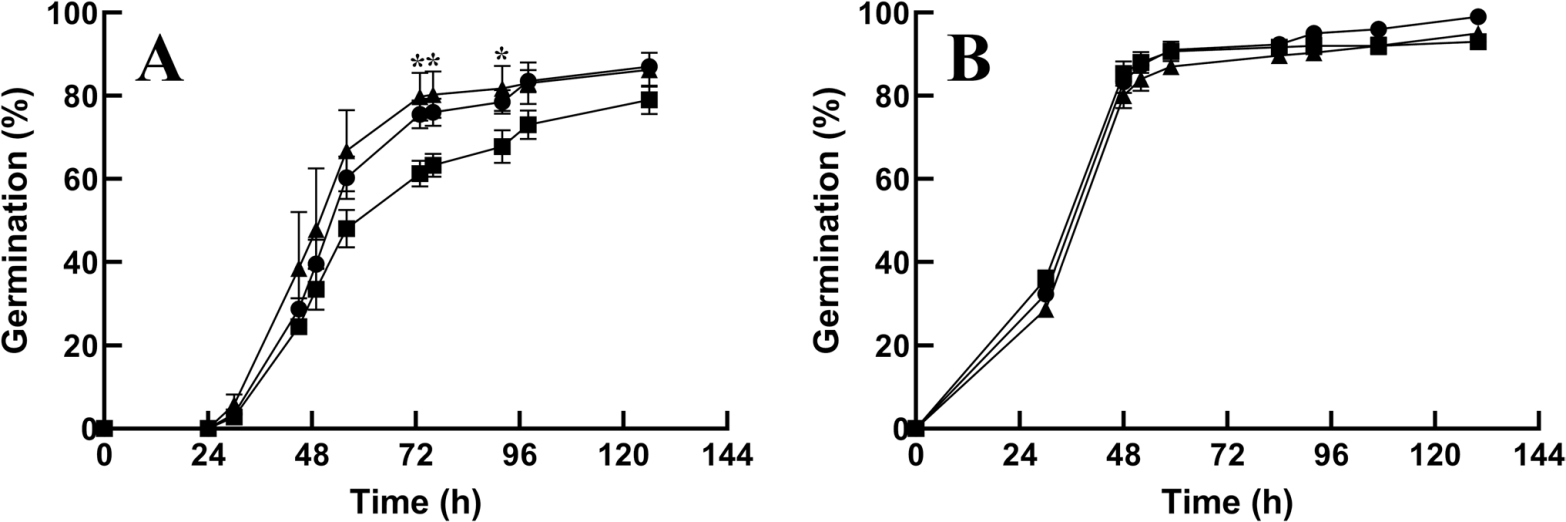
Germination of newly collected seeds. Freshly harvested, surface sterilized seeds of the wild-type (WT) (■), *pub41-1* (▲), and *pub41-2* (●) genotypes were plated on agar solidified 0.5 X MS 0.5% sucrose medium without pre-treatment (**A**) or after stratification (**B**). Germination was scored at the indicated times. Data represent means ± SE of three independent biological repeats. Asterisks mark significant differences between the WT and mutant seeds according to Student’s unpaired t-test (*p* < 0.05)

The rate of seed germination depends upon the speed of ABA degradation upon imbibition (Preston et al. 2009). Freshly collected Arabidopsis seeds display suppressed germination rates. Seed dormancy can be relieved with post-harvest storage time of dry seeds and can also be reversed by stratification of the seeds at any time point (Bewley et al. 2013). ABA is the primary hormone in the maintenance of dormancy, and gibberellins (GA) are the key players in germination (Gianinetti 2023). Thus, the ABA to GA ratio determines the fate of germination. Here we assayed germination using freshly collected non-stratified seeds. *pub41-1* and *pub41-2* seeds had higher germination rates compared with the corresponding wild-type seeds, indicating that in the absence of functional PUB41 there is a reduction in the ability to maintain dormancy (Fig. 6A). As expected, cold treatment of all tested genotypes broke dormancy and resulted in faster and similar kinetics of germination (Fig. 6B), supporting our interpretation that the differences in the germination of the non-treated seeds of the *pub41* mutants and wild-type plants result from reduced dormancy of the *pub41* mutants. Interestingly, a significant quantity of ABA is produced by a single layer of endosperm surrounding the embryo during germination (Karssen et al. 1983). This is compatible with the high *PUB41* promoter activity in embryos and endosperm we observed above (Fig. 2B, C). Moreover, mutation of the *AtPUB41* gene is expected to reduce plant fitness since reduced seed dormancy would result in early germination, threatening seedling survival during the harsh season, which is usually survived as a dormant seed that germinates only in environmental conditions favoring a completion of life cycle of the germinating seedling.

### ABA induces the expression of *AtPUB41*

**Fig. 7:**
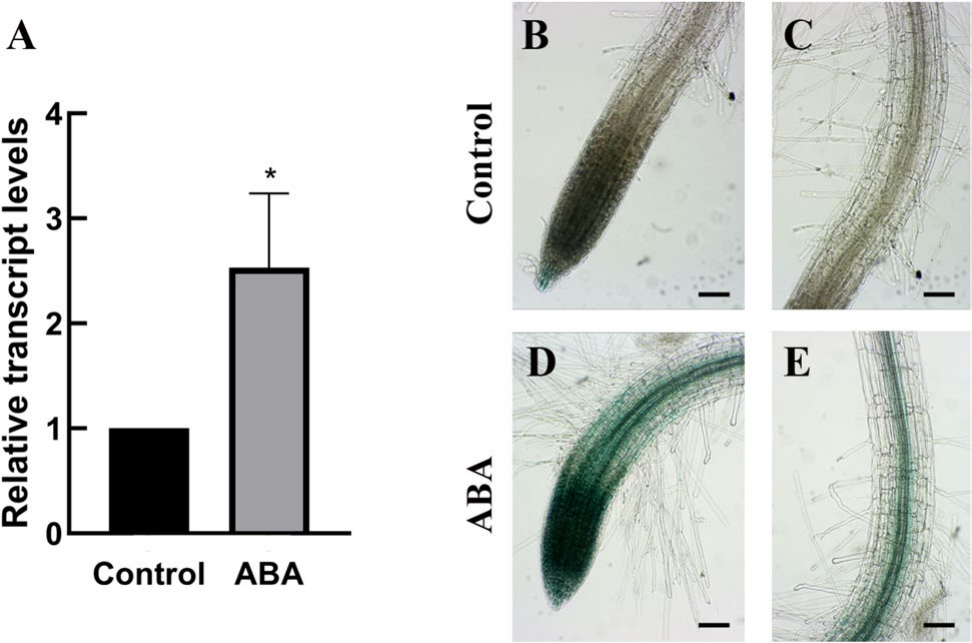
ABA induces *AtPUB41* expression. **A** RT-qPCR results showing *AtPUB41* transcript levels in roots of 10-days-old wild-type plants treated for 8 h without or with 10 µM ABA, using primers #12–14 (Table S1). Data show mean ± SE of three biological repeats. Asterisks denote statistical significance as determined by a two-tailed paired student’s t-test (*p* ≤ 0.05). **B**–**E** One-week-old *AtPUB41::GUS* Arabidopsis seedlings were treated for 8 h without (**B**, **C**) or with 10 µM ABA **D**, **E**, followed by histological staining for GUS activity. **B** & **D** primary root tips; **D**, **E** differentiation zone of the primary roots. Bars, 0.1 mm

Although the ABA signaling system has been well developed to modify the expression of a variety of stress-related genes (Rock 2000), the hormone does not affect the expression of genes involved in ABA sensing (for example, see Pardo-Hernández et al. 2024). We thus treated seedlings with ABA and assayed the effect on *AtPUB41* expression. Indeed, ABA treatment increases the steady-state levels of *AtPUB41* transcript in the root by 250% (Fig. 7A). In histochemical staining of plants expressing *AtPUB41::GUS* (Fig. 7B–E), we observed that ABA treatment enhanced *AtPUB41* promoter activity in the roots. Close to the root tip, *AtPUB41* promoter activity was present in all cell types whereas in the upper root zone it was primarily in the vascular system (Fig. 7D, E). Thus, *AtPUB41* behaves like a typical ABA-responsive gene whose expression is increased by this plant hormone.

### *AtPUB41* is a positive regulator of ABA-mediated seed dormancy and drought response

Our findings show that *AtPUB41* is a positive regulator of the ABA response. Germination of *pub41* mutants was less inhibited by ABA (Fig. 5) and the seeds displayed reduced dormancy (Fig. 6). ABA signaling consists of many proteins possessing different activities in the pathway and the high redundancy of some of these proteins, e.g., the ABA receptor (Yu et al. 2016). Positive regulation of ABA signaling by the E3 may result from degrading protein(s), which play a negative role in the signaling pathway (Yu et al. 2016). Thus, we propose that AtPUB41 substrates include negative regulators of ABA signaling located in the cytoplasm and/or nucleus, in agreement with the localization of AtPUB41 (Fig. 1). Most PUB genes involved in the plant response to environmental stress function as negative regulators of the plant stress response, i.e., the mutants are more resilient to stress than the WT, and overexpressing the respective genes often results in hypersensitivity (for recent reviews see Trenner et al. 2022; Trujillo 2018). In contrast, fewer PUBs act as positive regulators of the abiotic stress response: We previously showed that the paralogous genes *AtPUB46* and *AtPUB48* are positive regulators of the drought response, with single mutants being hypersensitive to drought (Adler et al. 2017). *AtPUB12* and *AtPUB13* act as positive regulators of ABA signaling as their E3 proteins degrade the ABA co-receptor ABI1 (Footitt et al. 2011). Furthermore, *AtPUB25* and *AtPUB26* positively regulate freezing tolerance (Wang et al. 2019), and *OsPUB67* is a positive regulator of drought tolerance (Qin et al. 2020).

PUB E3s have been implicated for their role in seed germination. *Atpub9*-KO mutants display hypersensitivity to ABA during seed germination (Samuel et al. 2008); suppression of the Arabidopsis *AtPUB30* results in decreased inhibition of germination by NaCl (Hwang et al. 2015); *AtPUB18* and *AtPUB19* genes are involved in salt inhibition of germination, with germination of double mutant plants being less sensitive to NaCl (Bergler and Hoth 2011).

*pub41* mutants are drought hypersensitive at the rosette stage and are less sensitive to ABA inhibition of germination than wild-type plants (Figs. 4 and 5). Similar phenotypes have been reported for other E3s, for example, single gene mutants of the paralogs Arabidopsis C3H2C3-type RING E3, *Arabidopsis ABA-insensitive RING protein* (*AIRP*) *AtAIRP1*, *AtAIRP2*, *AtAIRP3* (Ryu et al. 2010; Cho et al. 2011; Kim and Kim 2013) and *SALT- AND DROUGHT-INDUCED RING FINGER1* (*SDIR1*) (Zhang et al. 2007). In addition, mutants of the Arabidopsis F-box E3 ligases *ABA-RESPONSE KELCH PROTEIN 1* (*ARKP1*) (Li et al. 2016), *F-BOX OF FLOWERING 2* (*FOF2*) (Qu et al. 2020), *EID1-like protein 3* (*EDL3*) (Koops et al. 2011) also exhibit this phenotype. Like PUB41, all these genes, except SDIR1, are induced by ABA.

*AtPUB41* belongs to a 4 member-subfamily comprising *AtPUB38-AtPUB41* (Wiborg et al. 2008). *AtPUB40* was shown to mediate the root degradation of BZR1, a brassinosteroid-responsive transcription factor (Kim et al. 2019). Roots of the triple *pub39 pub40 pub41* mutant accumulated higher levels of BZR1 than WT. Unfortunately, these authors did not study the phenotype of the *Atpub41* single gene mutant, or *AtPUB41* overexpressor; thus, the role of *AtPUB41* in the brassinosteroid root signaling pathway remains unclear. Our data clearly show that *AtPUB41* is a positive regulator of ABA signaling, affecting seed germination and the drought response. The AtPUB41 target(s) within the ABA signaling pathway remain to be identified.

## Supplementary Information

Below is the link to the electronic supplementary material.

Supplementary file1 (PDF 32 KB)
