# Supplementary material for: Arabidopsis ubiquitin ligase PUB41 positively regulates ABA-mediated seed dormancy and drought response
Source: Physiol Mol Biol Plants. 2024 Nov 23;30(11):1819–27. doi: 10.1007/s12298-024-01526-6 (PMC11646239; doi:10.1007/s12298-024-01526-6)
Supplement: Supplementary file 1 — Supplementary file1 (PDF 32 KB) [file 12298_2024_1526_MOESM1_ESM.pdf]

Supplementary Table S1. Primers used in this study.

No.	Primer	Sequence	Application
1	PUB41 eGFP For	ACTACCATGGGTGGAAACAAGCAGAG	35S::PUB41-eGFP construct
2	PUB41 eGFP Rev	AACTGCAGAACTGGGAGGAATAAGCAAAAC	35S::PUB41-eGFP construct
3	LBb1.3	ATTTTGCCGATTTCGGAAC	Mutant verification
4	LP1_pub41-1	TCCATTTCGAAACGCTATGTC	Mutant verification
5	RP1_pub41-2	CTGGGAGGAATAAGCAAAACC	Mutant verification
6	LP1_pub41-2	CGATAGTGTCGCTCTATTCCG	Mutant verification
7	RP2_pub41-2	GTGGATTTCATTGCGAGATTG	Mutant verification
8	PUB41-FP1	TGTGTGGCGGTTCTGTTGAC	RT-qPCR (Mutant verification)
9	PUB41-RP1	CTT CGA CGC CTT CTC CTT CA	RT-qPCR (Mutant verification)
10	PUB41 Promoter For	AGTCTGCAGGTCCAATACTTTTAGTACTGTGC	Promoter cloning
11	PUB41 Promoter Rev	CTGGAATTCCGGCGAAAAAACTTGGG	Promoter cloning
12	PUB41-FP2	GAGCCGCTTCTTCTTTCTTCATC	RT-qPCR (WT ABA treatment)
13	PUB41-RP2	CGTTGGTGGAAAGAGAACCATC	RT-qPCR (WT ABA treatment)
14	ACTIN2-FP (AT3G18780)	TCCCTCAGCACATTCCAGCAGAT	RT-qPCR (reference gene)
15	ACTIN2-RP (AT3G18780)	AACGATTCCTGGACCTGCCTCATC	RT-qPCR (reference gene)
